# Future of the Voluntary Hospitals

**Published:** 1923-04

**Authors:** 


### THE FUTURE OF VOLUNTARY HOSPITALS.

THOSE who see in the continuance of the voluntary system the best hope for the future of the hospitals have every reason to take courage from the Report of the Voluntary Hospitals Commission, which is summarised in another part of our issue this month. The Report is, indeed, a gratifying indication of the resiliency of a system which, under stress of adverse circumstance, had seemed in danger of breaking down. It is true that, for the moment, the situation has been saved by the Government grant of half a million, but the experience of the Commission and the incentive to the hospitals to make special efforts to meet the grant has placed it beyond doubt that, given careful organisation and real co-ordination of effort, the hospitals can hold their own without embarking upon the "slippery slope" of aid either from the rates or the taxes. The proviso is, however, of essential importance. A large proportion of the community, certainly all hand workers and their employers, must be laid under systematic contribution in a way which the Hospital Saving Scheme in London and the many provincial schemes of a kindred character have already shown to be not only feasible but almost certain of success.

The Commission has done its work thoroughly. In accordance with the recommendation of Lord Cave's Commission, it has established Local Committees in nearly sixty provincial centres in England, Wales and Scotland. There is nothing amateur about these Committees. To a great extent they are composed of experts, not only full of zeal, but in many cases possessing initiative enough to raise new money from voluntary sources. We look upon these Local Committees as the sheet-anchor of the hospitals in the future. Local patriotism is one of the most potent influences upon endeavour of this kind. Fréderic Mistral said long ago that love of the village steeple is the foundation of patriotism, and recent events have shown that the cherishing of the town or district hospital is a much more powerful motive than many people had supposed. Its peril has put those who are proud of it on their mettle, and the Commission found that, generally speaking, the improvement in hospital finance is "very marked." No doubt much of the improvement is due to the lessened expenditure made possible by the fall of prices, yet a good deal of new money has been raised, and an entirely fresh standard of effort set up. It is probable that not nearly so much money would have been obtained had it not been for the much criticised pound for pound stipulation—a stipulation which the Government were clearly within their rights in making. The net result has been that the total of the new money raised has exceeded the amount of the grant. After much groping and the use of many expedients, it has now become clear to all the world that the care of the hospitals is everybody's business, and that the day of frenzied special appeals and of dependence upon large donations and legacies has gone by. We have got to be just as businesslike in this as in other and less important matters, and the Commissioners make a number of useful suggestions for placing finance upon a more satisfactory basis.

Great tasks are, however, still before us. For nearly ten years little has been done towards increasing hospital accommodation, and that accommodation is now more inadequate than ever. As population increases and disease is more and more controlled in its early stages, the pressure upon beds is certain to grow more severe. When means are available for the re-opening of closed wards and bringing back into use unoccupied beds, and hospitals can again cheerfully face their daily outgoings—and the fall in the cost of living will, to a considerable extent, be neutralised by the heavier demands of improved treatment—large schemes of extension will have to be taken in hand. We cannot stand still; we cannot look at long waiting-lists without making determined efforts to reduce the number of the poor and the suffering who are without hope save in the hospitals. The cost of building is unlikely ever to fall to its old level, yet without fresh building, and a great deal of it, the voluntary system must fail to meet the country's needs. For the hospitals to become a State service would be not only an economic but a spiritual calamity, since it would replace by a grudgingly paid tax that which at present is the spontaneous expression of duty and compassion. Yet we know that any serious failure to meet demands ever growing more insistent would be seized upon as a conclusive argument for saddling the State with the tribute which health and strength owe to those who are less blessed. In organising and systematising the collection of the people's pence, as well as the guineas of the more fortunate, we are treading the path of safety, and what those pence can do the Report of the Voluntary Hospitals Commission brings home to us once more with graphic force.

